# Book Reviews

**Published:** 1830-04

**Authors:** 


### CRITICAL ANALYSES.

Quæ laudanda forent, et quæ culpanda, vicissim

Illa, prius, cretâ; mox hæc, carbone, *notamus*.—Persius.

*Illustrations of some of the principal Diseases of the Ovaria, their Symptoms and Treatment; to which are prefixed, Observations on the Structure and Functions of these Parts in the Human Being and in Animals.* By Edw. J. Seymour, m.d., Fellow of the Royal College of Physicians of London, and one of the Physicians to St. George's Hospital. *With* 14 *Lithographic Engravings.* —8vo. pp. 126. London: Longman and Co. 1830.

Although many interesting facts have been ascertained respecting the structure and functions of the ovaria, we are as yet but infants in our knowledge of the nature of the various diseases to which these important organs are liable. To determine the existence of ovarial disease in its incipient stages is generally difficult, and sometimes impossible. When the malady is more fully developed, the difficulty of the diagnosis may perhaps be removed; but unfortunately our present means offer us but scanty hopes of eradicating it at an advanced period of its duration. We detect the enemy when we are no longer capable of presenting effectual opposition to his still further and fatal progress. Much, then, remains to be done before the subject to which Dr. Seymour especially directs our attention will be exhausted of its interest. The records of medicine bear ample testimony to the zeal with which it has been investigated; and in the present day there is too much ardour and humanity in our profession to stop short in an inquiry of such vast importance. The object of the work before us is to lay before the reader the principal part of what is known in the history of ovarial diseases, the appearances presented on dissection, the remedies which have been proposed for the entire cure, and the palliative means which diminish the sufferings of the patients, and render life endurable.

In the first chapter, Dr. Seymour traces the outline of the functions of the ovaria in the various classes of animals, especially the vertebrated animals; as in many instances what is obscure, or altogether unknown, in one class, is rendered more clear by the more simple contrivance or more visible structure adapted for similar purposes in another.* That physiology should be considered the basis of all true pathology, we are quite convinced; and we are gratified to find that Dr. Seymour considers "it is hopeless to expect that any considerable improvement should be made in the treatment of the various and complicated diseases of the ovaria in the human being, unless the structure and functions of these parts are fully understood; and that, without this knowledge, we shall be forced to resign to their fate the unfortunate victims of these unsightly and often painful diseases, with a sigh for the insufficiency of our art." It is not asserted that even when these laws and these structures are clearly defined, much will not be left to be effected in the treatment of ovarial diseases, much to be referred to individual constitution, to the artificial laws which regulate civilized society; more to be hoped for from the discovery of new remedies, or the application of those already known, by ingenuity and talents.

We shall confine ourselves, in our notice of the first chapter, to the chief conclusions Dr. Seymour draws from his own observations, and the assistance he has derived from his researches as to the structure and functions of the ovaria in animals and the human female.

"In the class of birds,† the ovarium consists of a number of rounded or oval bags, each attached by a peduncle to a common stalk, and termed, from their contents, the yolk or yelk bags. It is placed immediately above the kidneys, at the bifurcation of the aorta. It is enclosed in peritoneum, which connects it with the spine. Immediately below opens a tube similar in appearance to an intestine enveloped in peritoneum, which fixes it to the spine, and likewise restrains its mobility. This tube is the oviduct: the extremity near the ovarium is free in the abdomen, is of great tenuity and transparency, and has received the name of infundibulum. As the duct descends it becomes thicker, and towards the inferior part a few muscular fibres are visible. The interior of this tube is covered with villi, from which is secreted first the white, and afterwards, at the inferior parts, the shell. These villi do not apparently differ in structure, except that in the portion which secretes the white, they appear to have a longitudinal, in the portion where the shell is perfected, a transverse arrangement." (P. 6.)

The oviduct performs the same office to the ovarium which is effected by the fallopian tubes to the ovaria of the

* From the study of the structure of the ovaria in animals, says Cuvier, some of the most brilliant results of comparative anatomy have been derived. —Rev.

† Plate 1, fig. 1 and 2.

mammalia: it is certainly the structure through which the ovum passes.

"At the period when a yelk bag is matured, a white line is formed across the centre of the capsule: this white line is from the absence of vessels; they become obliterated at that part, and this is well seen in injected specimens. In the direction of the white line, the capsule bursts; the yelk bag is grasped by the extremity of the oviduct, which appears endowed with a contractility *sui generis;* and being propelled downwards by a motion probably similar to the peristaltic, receives, as has been already stated, the white and shell." (P. 7.)

The structure of the oviduct, and its relative position to the ovarium, is the same in all the genera of birds, as far as Dr. Seymour's observation has enabled him to determine.

"It has only occurred to me to observe the parts in question in the crocodile, in the unimpregnated state.* There are two ovaria and two oviducts, the latter resembling perfectly the same organ in birds. The infundibula were turned outwards, which renders it a matter of more than usual difficulty to account for the way in which the ova enter the oviduct. It is not improbable that a considerable change takes place in the relative position of parts when the animal is impregnated. The oviducts terminate on each side in the cloaca." (P. 10.)

Many curious and interesting facts are stated respecting the structure and functions of the ovaria in various other animals. Having sketched out, in the different classes of vertebrated animals, the structure of the ova which contain the germ, we find that in fish, the amphibia, and birds, they exist previously to impregnation; when fecundated, pass into a tube, where they receive the coverings necessary to protect them from external violence, until the young animal is ready to be ushered into the world.

"To carry this analogy to the mammalia, and subsequently to the human being, is easy: the Graafian vesicles present the greatest resemblance to the ova of other animals; they become larger, more turgid, and more vascular, at puberty. After impregnation, fissures are observable in the external coat of the ovarium, and a hollow space is visible in its substance, once filled by the vesicle round which a new body is formed: all this can be demonstrated, and can be seen by any observer." (P. 15.)

These circumstances led to the opinion, in which De Graaf, and all the eminent physiologists who immediately succeeded him, participated, that the Graafian vesicles were ova, similar, in the human species and the mammalia, to what the ova in birds, reptiles, and fish were to their

* Plate 2.

respective kinds. By some distinguished physiologists, especially of the Italian school, this opinion was opposed. It was said, if the corpora Graafiana were really ova, they should be detected in their passage through the fallopian tube; and in some animals where they are very large, and the canal extremely small, such passage would be impossible. De Graaf asserted that he saw the ova in the fallopian tube; but neither Hartmann, Valisneri, nor Haller, in numerous experiments for the purpose, ever succeeded in seeing the vesicle or ovum in the fallopian tubes or uterus. Since this period, however, they were distinctly seen by Haighton and Cruickshank, who confirmed De Graaf's experiments. Here, however, arose a fresh difficulty: the ova thus seen were greatly smaller than the unimpregnated Graafian vesicle.

"Satisfied, from all the observation it has been in my power to make, that the original opinion is the correct one, the difficulties may be reconciled from considering that the ova in the different classes do not contain similarly arranged matter. Thus the yelk bags in birds, amphibia, and fish, contain not only the germ of the future fœtus, but a considerable portion of aliment for its subsistence: hence they bear a much larger proportion to the animal than the corpus Graafianium to man; but in the mammalia no such provision is necessary. The more perfect being requires a longer period for maturity, and a different arrangement for its nourishment, and this is provided in the uterus; hence the Graafian vesicle contains probably only sufficient fluid to protect the germ from injury, and, when grasped by the fallopian tube, the contents of the vesicle are discharged, and the rudiment, not the entire vesicle, carried to the uterus. Such is the fair conclusion, and not that the laws of nature, so uniformly and beautifully adapted to the end in view, are changed, and an entirely new process, untraceable to the senses, substituted in their place." (P. 16.)

The experiments of Baer prove, indeed, to demonstration that such conclusions are correct. He has seen the ovum, contained in the vesicle in the ovarium, exactly similar to those which he (as well as Haighton and Cruickshank) found in the fallopian tube. Many conflicting opinions have been maintained upon the question of whether the fallopian tubes in the mammalia, and the oviducts in the other vertebrated animals, convey the seminal fluid to the ovum. The numerous experiments on record, and all the comparisons he has been enabled to make between the process in various animals, lead Dr. Seymour to the conclusion "that the fallopian tubes convey the fecundating fluid to the ova, and are the media through which the ova pass to the uterus in more perfect animals, and prepare them for the process of incubation in the less perfect."

A brief sketch is given of the discussions which have arisen respecting the formation of the corpora lutea. One of the latest authors on this subject is Sir Everard Home, whose "views entirely accord with those of Malpighi, Valisneri, &c., although he does not allude either to their opinions or experiments." That corpora lutea exist occasionally without impregnation, must be admitted by all. That they occur after impregnation is certain, and proved by the observations of Haller, who traces their gradual formation. If, as supposed by Sir E. Home, they are necessary to render the ovum fit for impregnation, they should exist nearly always in virgin animals at the time of puberty.

"This is by no means the case. It has occurred to me to have examined the ovaria in the human being and in animals at the period of puberty, in very many instances: many had ova ready for impregnation, large, projecting, vascular, yet no corpora lutea were visible, which induces the following conclusion, that in every instance these animals must have been barren, or that the formation of corpora lutea is not a necessary preliminary process to impregnation.

"From these premises, comparisons, and observations, my opinion has been formed, that corpora lutea are the result of the change which takes place in the ovarium by the bursting and discharge of the ovum, occurring rarely in virgin animals, because the bursting of the ovum is not a frequent, but only possible occurrence, but always following impregnation, and diminishing as gestation proceeds." (P. 32.)

The corpora lutea are the vascular remains of the Graafian vesicle, after its rupture and the discharge of its contents. It is believed that in human beings certain feelings of the mind are sufficient to determine the rupture of the vesicle. In birds, an egg passing into the oviduct, the membrane which formerly contained it, shrinks; but this occurrence takes place equally whether the ovum which so passes be or be not impregnated. In order to prove that such an effect may occur from excited feelings, eggs have been produced from birds by impressions calculated to promote such feelings, without the presence of the male bird. Haller believed that the corpus luteum was the product of conception alone. The opinion of this great physiologist was opposed by Buffon, Bertrandi, Valisneri, and other Italian anatomists, who assert that they had found corpora lutea in the ovaries of many virgins; and according to the observations of the ingenious Blumenbach, the excitement of solitary sensuality, or of a barren marriage, may equally give rise to the formation of these productions. Brugnoni, also, has stated several anatomical facts,* which show that the corpora lutea may exist before impregnation.

"It may here be asked, of what advantage is it to determine accurately the formation of these bodies? We have seen that their production is probably influenced by strong moral as well as physical impressions, the result of great vascular excitement of the part, and their absorption effected by great activity in the vessels of that system. Any deficiency, then, in the quantity of vascular excitement necessary, any obstacle to the exercise of absorption, would produce changes in these parts differing from the natural ones which they were intended to undergo, would, in a word, produce disease; and it remains to be discovered whether any of the serious and complicated diseases of these organs are to be traced to alterations which the corpora lutea undergo from any or all of these causes." (P. 33.)

Dr. Seymour observes, that in the human being, in extremely rare cases, one ovarium has been found wanting, yet the female has borne children; and one being destroyed by disease has repeatedly been found to be no prevention against the female becoming a mother, from ova formed in the remaining organ. The knowledge of rare deviations from the natural structure can be but of little importance to the practitioner; but we may just observe that Morgagni (lib. i. p. 12, 13,) states that he had found both ovaries wanting. The same fact was ascertained some years ago at the Hospice de la Maternité at Paris. Poupart found in the body of a girl, seven years of age, that the left ovary had neither spermatic artery or vein.

Chapter II. "On the Diseases of Structure in the Ovaria." These diseases are various: they may arise from inflammation of the structures of which the ovarium is composed; from enlargement of the natural structure, or from the addition of new structure formed by disease, including scirrhous and fungoid growths, which, from their rapid progress, their assimilation of neighbouring structures, their coincidence with other cancerous diseases in the same patients, and their fatal tendency scarcely admitting of palliation, have received the appellation of malignant growths. Those deviations from natural structure which arise from obstruction in the function they are destined to perform, and those alterations of them which are probably congenital. The ovaria, like every other part of the body, are liable to inflammation.

* La Médecine éclairée par les Sciences Physiques, par Fourcroy, t. ii. 142.—Rev.

"It does not, however, appear that inflammation of the peritoneal covering of the ovaria takes place without general inflammation of that portion of the membrane which covers the fundus of the uterus, and, if it does, is not discoverable by any known or definite symptoms. No particular sensibility is increased, no particular sympathy with distant parts excited; nor can the remedies, therefore, be distinct from those which apply to inflammation of the uterus generally." (P. 38.)

Inflammation of an acute form may attack the substance of the ovarium, from which arises suppuration or softening of the part. These forms of disease usually depend upon acute inflammation of the womb, and its appendages, in women after labour. Chronic inflammation of the ovarium may exist independently of inflammation of the substance of the uterus or its coverings. Abscess of the ovarium Dr. Seymour states to be a rare disease: he describes one case of this kind. We may observe, that Portal (Anatomie Médicale) had seen the ovaria full of pus, and larger than the head of an infant. Chambon (Traité sur les Maladies des Femmes) also had frequently known inflammation of the ovaria terminate by suppuration. The pus has been discharged by the bladder. We quote the following curious case as an example, which was communicated in 1753 to the Académie Royale de Chirurgie.

A lady had complained for a long time of severe pains in the right lumbar region. Pus was discharged with the urine, and it was supposed that the right kidney was diseased. The patient died, and the kidney was found to be healthy; but the ovarium of that side adhered to the fundus of the bladder, where there was an opening which penetrated to the ovarium, and thus the pus passed into the bladder.

"Whether the Graafian vesicles are ever affected by inflammation, except when in common with the substance of the ovarium, it would be impossible to determine, except by long-continued and very accurate examination after death." (P. 41.) Dr. Seymour thinks it would be still more difficult to say what is or what would be the effect of inflammation of the corpora lutea; that is, of vascular excitement greater than what is necessary for their formation. The fluid which is contained in the Graafian vesicles is liable to disease: it is often red, and even black, from the admixture of blood; and it appears to Dr. S., from a case which he relates, that it may become altered from imperfect fecundation.

"By far the most frequent disease of the ovarium, and of course that which occurs to our observation in practice, is the conversion of this organ into numerous cysts, of various sizes; or the production of similar cysts, having their origin in some part of the ovarium; and when either the whole or some of these cysts contain fluid, the disease has received the name of ovarian or encysted dropsy. Under the name of ovarian dropsy have also been included simple serous cysts, formed in the broad ligaments and fallopian tubes.* All these, confounded together under the name of hydatids, are distinguished from the latter by being nourished by vessels supplying them from the parts in which they are formed; vesicles to which the name hydatid is attached being nourished by their own blood-vessels, or, in other words, having an independent life. Occasionally one or both ovaria are converted into simple cysts; the whole of the cellular substance and vesicles disappearing, that which was the fibrous coat of the ovarium becoming the fibrous coat of the cyst.

"The first form of this disease, and the simplest, is from an enlargement or alteration of the corpora Graafiana. At an advanced period of life, on cutting into the ovarium, one or more of the Graafian vesicles are found dilated; and these bodies, generally the size of a millet seed, become as large as an almond, are filled with limpid fluid, and their internal membrane becomes very vascular. Such is a common appearance; but occasionally they enlarge to a greater degree, and always on the side nearest the proper coat, which becomes distended often to an enormous size.† In this way it appears to me that a large single cyst with a fibrous covering may be formed; and this is the simplest form of ovarian dropsy, the internal membrane secreting a prodigious quantity of fluid. The same opinion is entertained by Cruveilhier, and expressed in his work on Morbid Anatomy, still in progress of publication. His words are these: 'L'ovaire est converti en une poche unique, qui peut acquérir un volume tel qu'elle remplisse la presque totalité de l'abdomen, à la manière d'une ascite. Il est probable que dans ce cas une seule vesicule aura par son developpement effacé le reste de l'organe qu'on rencontre atrophié sur l'un des points de la circonférence de la poche, et confondu avec des épaississements cartilagineux et osseux.' One or two of the Graafian vesicles undergo this change, when the disease consists of one or two thin cysts filled with fluid." (P. 44.)

Encysted ovarial dropsy may exist for years without much distress, and may furnish, by paracentesis, a wonderful quantity of fluid. A patient of Mr. Keate's, in St. George's Hospital, in 1828, was tapped four times in three years, and seventy-five pints of fluid were withdrawn:

* Plate 8.

† "A similar disease affects the ovarium in birds, one or more of the yelk bags becoming enlarged and distended by the accumulation of transparent white fluid. It has been often seen in domestic fowls."

she is now alive. This quantity, however, is trifling in comparison with many well-attested cases on record.

"The ordinary symptoms attendant on ovarian dropsy are very various, and by no means severe, and are limited principally to the effects of pressure on the neighbouring parts. Where the increase of the disease is slow, the patient often suffers no other inconvenience than from swelling of the leg on the side on which the tumor is largest, or from the unsightly bulk of the abdomen, which she is unable to conceal. Patients have lived in this manner thirty or forty years, with a very considerable enjoyment of the comforts of life, and even the pleasures of the world,* the accumulation of fluid rendering it necessary from time to time to perform the operation of paracentesis. In cases of this kind, symptoms dependent on unusually rapid increase of bulk, or pressure on any particular organs in the abdomen, occur. Thus heartburn, vomiting, and purging, difficulty of passing urine, or violent and severe headach, are met with, which are entirely removed if the bulk of the tumor be reduced. There is a case now under the care of Mr. North, where the patient has for many years been unable to pass her urine, except by the daily use of the catheter; and this appears to arise from the natural situation of the bladder being altered by pressure, and perhaps by the adhesion of the tumor." (P. 48.)

Various opinions have been entertained by the most eminent practitioners as to the nature of the case here alluded to. We agree perfectly with Dr. Seymour, who was kind enough to give his opinion, that the disease is encysted ovarial dropsy. The origin, progress, and attendant symptoms of the case appear to show that such is the fact. Dr. Macleod is also of this opinion. The chief distress which this lady suffers is from the excessive bulk of the tumor. She menstruates with difficulty and pain, and the discharge is very small in quantity. There is one circumstance respecting this case which appears remarkable: for upwards of five years we were obliged to introduce the catheter twice daily, with the exception of about a dozen times, when the usual quantity of water was passed without assistance. It is curious that the bladder should still retain its expulsive power, when so seldom called into action. The patient now remains in the same state, and still requires the daily use of the catheter, in the introduction of which some adroitness is required, in consequence of the

* One of the most remarkable instances of this is related by M. Frank. "A case is known of a girl affected with ovarian dropsy at thirteen years of age, having lived to the age of eighty-eight, notwithstanding the size of the tumor, which occupied the whole of the lower belly."—*Médecine Pratique de Pierre Frank*, p. 236.

pressure of the tumor upon the neck of the bladder. The surgeon who was first consulted failed in his attempts, and the second occupied upwards of two hours in the operation. We may be allowed some self-gratification for having succeeded with little or no difficulty.

When both ovaria are diseased in this way, Dr. Seymour states that the catamenia are always absent; when only one ovarium is affected, they are sometimes irregular, sometimes absent altogether.

The diagnosis of this disease is sometimes difficult. The distinctions between ovarial dropsy and ascites are pointed out: they are often, however, very obscure, and can hardly lead to a definite opinion as to the nature of the case. In general, ovarian dropsy continues throughout the whole course of life. It sometimes, however, disappears under very remarkable circumstances; either by adhesion being formed between the tumor and some portion of the great intestine, the matter being passed by stool. In such cases the patient often recovers. The fluid has been discharged by the vagina. Occasionally, after adhesion between the cyst and the parietes of the abdomen, spontaneous rupture takes place at the umbilicus, and the contents of the cyst are discharged, and the patient survives.

"Through the kindness of Dr. Locock, a case in which an attempt at a termination of this kind occurred came under my observation last autumn. A woman, of about fifty years of age, had suffered for several years from an enormous encysted dropsy, springing apparently from the right ovarium: a fistulous sinus had formed at the right of the umbilicus, from which the fluid constantly dripped when any increase of the secretion greater than ordinary occurred; and thus the distention of the patient, otherwise unendurable, was relieved." (P. 53.)

Dr. Blundell relates the following case in his lectures. A lady afflicted with this disease, falling from a carriage, struck her belly against a stone; a considerable discharge of urine occurred. She recovered, married, and, dying subsequently of retroversion of the uterus, the cyst of her former complaint was found to have burst, and its contents, effused into the abdominal cavity, to have been absorbed. Rupture of the cyst, however, must always be considered highly dangerous.

Dr. Seymour refers to the experiments of Magendie and Blundell, which prove that bland fluids may be injected into the peritoneal and pleural cavities of animals, without necessarily inducing death; and he observes, that the albuminous fluid contained in some ovarian cysts may be supposed to be little irritating. The whole of the ovaria have been said to be sometimes converted into bone. Such cases Dr. Seymour has never seen, and the various museums of morbid anatomy in this city contain no such examples.* Scrofulous disease occasionally attacks the ovaria, especially in young girls.

Scirrhus, and malignant or fungoid diseases of the ovarium, are next described, of both which forms of disease cases are detailed, to point out their progress and effect upon the constitution of the patient.

"One of the most frequent morbid appearances connected with the ovarium, arises from tumors either in the substance of the ovarium or attached to it by a peduncle, containing hair and adipose matter, and imbedded in some portion of the mass or the fibrous membrane which surrounds it: a portion of bone is often observed, sometimes part of an alveolar process containing one or more teeth, and occasionally a well-formed jaw with perfect teeth." (P. 82.)

There are numerous and well-authenticated cases of this kind on record. In what manner these growths are produced in the ovaria, still remains doubtful. Dr. Seymour believes that they are the result of an imperfect conception in the mother of the individuals in whom they are found.

Fœtuses are occasionally developed in the ovarium. This occurrence cannot happen without in some measure constituting a disease of the part. The natural form and texture of the ovarium must be altered, as well as the vital properties of the organ. This species of pregnancy, also, in most cases, gives rise to serious symptoms, which lead to the destruction of the infant and the mother.

Chapter III. "Treatment of Diseases of the Ovarium." Inflammation of the acute form attacking the ovarium seems best relieved by local depletion, as cupping on the loins and sacrum; the tepid bath, opiates, rest, and a recumbent posture. Where abscess in the ovarium adheres to the adjacent parts, the patient's strength must be supported by bark, light nourishing diet, and mild aperients. Rest and pure air are here of essential service. Scrofulous disease of the ovaria, Dr. Seymour apprehends, seldom occurs without symptoms of a similar disease in other organs of the body. Remedies which invigorate the health, pure air, nourishing diet, a mild and equal climate, and alkaline medicines are recommended.

* In the Dict. des Sc. Med. t. 39, p. 21, a case of this kind is referred to. —Rev.

*Simple encysted Dropsy.* It does not appear that the quantity of fluid contained in the cyst can be diminished by diuretic remedies; but the disease is not unfrequently combined with effusion into the peritoneum, and then the bulk and distress of the patient are greatly relieved by diuretics. Of these, the most powerful which have occurred to Dr. Seymour are infusions of digitalis and of the pyrola umbellata. Emetics have been employed, from their known power of promoting absorption. Bloodletting and mercurials are often important auxiliaries. Long-continued frictions have appeared to be very useful, the dropsy having gradually and entirely disappeared under such treatment. A case of this kind occurred to Dr. C. M. Clarke. Upon the subject of tapping Dr. Seymour observes, that "very many times life has been protracted by the operation many years, and probably in this, as in many other cases, it is desirable to avoid extremes; not to have recourse to paracentesis earlier than appears necessary, on the one hand, nor to let the patient languish in unendurable distention on the other, from a vain fear, which at last may not be realized, of the rapid re-collection of the fluid." Two methods have been proposed for emptying the cyst, and for promoting its entire contraction.

"1. A considerable incision, in order to empty the cyst entirely of its contents, leaving in a canula or bougie, to excite contraction of the cyst, and prevent the re-collection of fluid.

"2. Injections into the cyst." (P. 99.)

In a few cases, both these modes of treatment have succeeded, but they cannot be recommended with much confidence.

"Leaving in the canula, or a bougie, after paracentesis, has been frequently tried. I am indebted to my friend Mr. Key, senior surgeon to Guy's Hospital, for a note of three cases in which he employed this practice; and, as it has failed in the hands of this scientific and accomplished surgeon, I fear it is not likely ever to be attended with the success which would establish its general employment." (P. 103.)

Dr. Seymour candidly confesses that, in true scirrhous and malignant diseases of the ovarium, he is unable to propose any remedy which can be much relied on. Mercury, iodine, caustic, alkali, conium, and muriate of lime, have each been supposed to cause the removal of these morbid growths, but to be more effectual in recent cases, where the tumors are soft and spongy in their texture, than in those of a solid or fibrous character. Upon each of these remedies Dr. S. offers many instructive practical observations. The efficacy of iodine, he thinks, has been greatly overrated. In diseases of a malignant nature affecting internal parts, the Liquor Potassæ, in as large doses as the stomach will bear, has appeared to Dr. Seymour to have produced more alleviation than any other remedy with which he is acquainted. In ovarian disease, of the kind now under consideration, it has appeared to be useful. Conium does no more than relieve pain. Repeated blisters have been recommended by Mr. Abernethy, but Dr. S. has little confidence in this practice. Muriate of lime is said to be inferior to the Liquor Potassæ, as far as the observation of the author extends. Dr. James Hamilton, however, strongly recommends it; but he conjoined with it percussion of the tumor, and he states that, in seven cases where this plan was adopted, the enlargement so completely subsided that it was no longer tangible. The last measure for the cure of this disease, and of which in modern times we have heard much, is the extirpation of the whole tumor. This operation has been recently successfully performed several times on the continent, and in our own country by Mr. Lizars* of Edinburgh. "But the arguments against such an operation are numerous and strong, and the probabilities of success are very small."

The subject of hernia of the ovarium is not touched upon. This disease is by no means common, nor is it easily distinguished from some others. It has even been confounded with enlargement of the inguinal glands. A celebrated French practitioner, M. Deneux, has collected all the published cases of hernia of the ovaria, and an interesting abstract of his work is given in the Dict. des Sciences Med. t. xxxix. p. 34.

To both medical and surgical practitioners Dr. Seymour's work must be highly interesting. He has given, in a concise form, a very perspicuous and instructive account of the information we possess relative to the structure, functions, and diseases of the ovaria, together with their treatment. The skill he has evinced in his selections from the materials of previous inquirers, and still more his original observations, prove that he possesses a sound practical knowledge of his subject. The low price of the work bears testimony also to his liberality and disinterestedness.

The accompanying lithographic engravings are beautifully executed, and without their assistance the work would have been much less useful.

* See Mr. Lizar's work on Operations for Diseased Ovaria.

*Elements of Physics, or Natural Philosophy, General and Medical, explained independently of technical Mathematics.* In two Volumes. Vol. II. Part I. *comprehending the Subjects of Heat and Light.* By Neil Arnott, M. D. of the Royal College of Physicians.—8vo. pp. 320. London: Longman, and Underwood, 1829.

We derived so much amusement and instruction from the first volume of Dr. Arnott's Elements of Natural Philosophy, that we must confess ourselves somewhat to blame for not having before directed the attention of our readers to the present continuation of the work, in which are comprehended the subjects of "Heat and Light." The second part of the volume, comprising the subjects of "Electricity, Magnetism, and Astronomy," and concluding the work, will, we believe, speedily be published.

In the fourth edition of the first volume, Dr. Arnott gave a complete explanation of the hitherto unknown nature of the defect called *stuttering* or *stammering;* "and the discovery of its nature has suggested to the author an effectual remedy, so simple that sufferers in general will be able at once to adopt it from the description now given." That the purchasers of former editions may not be obliged to procure the last on this account alone, Dr. A. very liberally prefixes to this volume an appendix to the early editions; and as it is brief, and of much practical importance, we give it in his own words:

"The most common case of stuttering is not, as has been almost universally believed, where the individual has a difficulty in respect to some particular letter or articulation, by the disobedience, to the will or power of association, of the parts of the mouth which should form it, but where the spasmodic interruption occurs altogether behind or beyond the mouth, viz. in the glottis, so as to affect all the articulations equally. To a person ignorant of anatomy, and therefore knowing not what or where the glottis is, it may be sufficient explanation to say, that it is the slit or narrow opening at the top of the windpipe by which the air passes to and from the lungs, being situated just behind the root of the tongue. It is that which is felt to close suddenly in hiccup, arresting the ingress of air, and that which closes, to prevent the egress of air from the chest of a person lifting a heavy weight, or making any straining exertion; it is that, also, by the repeated shutting of which a person divides the sound in pronouncing several times, in distinct and rapid succession any vowel, as o, o, o, o. Now, the glottis during common speech need never be closed, and a stutterer is instantly cured if, by having his attention properly directed to it, he can keep it open. Had the edges or thin lips of the glottis been visible, like the external lips of the mouth, the nature of stuttering would not so long have remained a mystery, and the effort necessary to the cure would have forced itself upon the attention of the most careless observer; but, because hidden, and professional men had not detected in how far they were concerned, and the patient himself had only a vague feeling of some difficulty, which, after straining, grimace, gesticulation, and sometimes almost general convulsion of the body, gave way, the uncertainty with respect to the subject has remained. Even many persons who, by attention and much labour, had overcome the defect in themselves, as Demosthenes did, have not been able to describe to others the nature of their efforts, so as to ensure imitation; and the author doubts much whether the quacks who have succeeded in relieving many cases, but in many also have failed, or have given only temporary relief, really understood what precise end in the action of the organs their imperfect directions were accomplishing.

"Now a stutterer, understanding of anatomy only what is stated above, will comprehend what he is to aim at, by being further told that, when any sound is continuing, as when he is humming a single note or a tune, the glottis is necessarily open, and, therefore, that when he chooses to begin pronouncing or droning any simple sound, as the *e* of the English word *berry*, (to do which at once no stutterer has difficulty,) he thereby opens the glottis, and renders the pronunciation of any other sound easy. If then, in speaking or reading, he joins his words together, as if each phrase formed but one long word, or nearly as a person joins them in singing, (and this may be done without its being at all noted as a peculiarity of speech, for all persons do it more or less in their ordinary conversation,) the voice never stops, the glottis never closes, and there is of course no stutter. The author has given this explanation or lesson, with an example, to a person who before would have required half an hour to read a page, but who immediately after read it almost as smoothly as it was possible for any one to do; and who then, on transferring the lesson to the speech, by continued practice and attention, obtained the same facility with respect to it. There are many persons, not accounted peculiar in their speech, who, in seeking words to express themselves, often rest long between them on the simple sound of *e* mentioned above, saying, for instance, hesitatingly, 'e I e........ think e........you may;' the sound never ceasing until the end of the phrase, however long the person may require to pronounce it. Now a stutterer, who, to open his glottis at the beginning of a phrase, or to open it in the middle after any interruption, uses such a sound, would not even at first be more remarkable than a drawling speaker, and he would only require to drawl for a little while, until practice facilitated his command of the other sounds. Although producing the simple sound which we call the *e* of *berry*, or of the French words *de* or *que*, is a means of opening the glottis, which by stutterers is found very generally to answer, there are many cases in which other means are more suitable, as the intelligent preceptor soon discovers. Were it possible to divide the nerves of the muscles which close the glottis, without at the same time destroying the faculty of producing voice, such an operation would be the most immediate and certain cure of stuttering; and the loss of the faculty of closing the glottis would be of no moment.

"The view given above of the nature of stuttering and its cure, explains the following facts, which to many persons have hitherto appeared extraordinary. Stutterers often can sing well, and without the least interruption; for, the tune being continued, the glottis does not close. Many stutterers can also read poetry well, or any declamatory composition, in which the uninterrupted tone is almost as remarkable as in singing. The cause of stuttering being so simple as above described, one rule given and explained may, in certain cases, instantly cure the defect, however aggravated, as has been observed in not a few instances; and this explains also why an ignorant pretender may occasionally succeed in curing, by giving a rule of which he knows not the reason, and which he cannot modify to the peculiarities of other cases. The same view of the subject explains why the speech of a stutterer has been correctly compared to the escape of liquid from a bottle with a long narrow neck, coming 'either as a hurried gush or not at all;' for, when the glottis is once opened, and the stutterer feels that he has the power of utterance, he is glad to hurry out as many words as he can, before the interruption again occurs.

"Should the author's future experience enable him to simplify or render more complete the views of the nature and cure of stuttering which he has given above, so as to facilitate the cure in every variety of case, he will not fail to publish his remarks." (P. v.)

We cannot enter into a formal analysis of the immediate subjects of the volume before us. Our object is merely to do justice to Dr. Arnott, by strongly recommending the perusal and study of it. It is the duty of every medical student to acquire at least an elementary knowledge of such important subjects as heat and light, both of which involve many points that are intimately connected with his profession. It is his interest also, because it is impossible he can mix in the society of men of liberal education without sometime or other hearing these subjects discussed, and if he is found to be unacquainted with them, a disadvantageous comparison may very probably be drawn between his philosophic and professional information. The great merit of Dr. Arnott's style, and mode of illustrating even abstruse problems, is simplicity. In his work we have not to lament the dry and repulsive manner, and the incessant recurrence of mathematical perplexities, which absolutely frighten the junior student, and not unfrequently puzzle the inquirer of maturer age: his "Elements of Physics" may be taken up when the mind is fatigued with more severe labours; and if the student will occasionally bestow an hour in the perusal of it, he will quickly find himself in possession of much useful and highly interesting information, at half the expense of time and mind that would be required if he consulted other works upon the same subjects. The utility of the work is also much increased by the numerous illustrative diagrams which are given in various pages.

The first part of the second volume is divided into two sections: the first section is "on Heat," and from it we quote the following passage.

" *The Functions of Animal Life a Source of Heat.*

" It is one of the remarkable facts in nature, that living animal bodies, and to a certain degree living vegetables also, have the property of maintaining in themselves a peculiar temperature, whether surrounded by bodies that are hotter or colder than they. Captain Parry's sailors, during the polar winter, where they were breathing air that could freeze mercury, still had the natural warmth in them of 98° of Fahrenheit; and the inhabitants of India, where the same thermometer stands sometimes at 115° in the shade, have their blood at no higher a temperature.

" It was at one time the favorite explanation of this, that animal heat was produced in the lungs, during respiration, from the oxygen then admitted. This oxygen combines with carbon from the blood, and becomes carbonic acid as in combustion, and it was supposed to give out a portion of its latent heat, as in actual combustion; which heat being then spread over the body by the circulating blood, maintained the temperature. We now know that if such a process assist, which it probably does, (for the animal heat has generally a relation to the quantity of oxygen expended in any particular case, and when an animal, being already much heated, needs no increase, very little oxygen disappears,) still much of the effect is dependent on the influence of the nerves, either directly or indirectly through the vital functions governed by them. Mr. Brodie, in his important experiments upon the subject, found that, although in animals apparently dead from injury done to the nervous system, he could artificially continue the action of respiration, with the usual formation of carbonic acid, still the temperature fell very quickly. The maintenance of low temperature in an animal immersed in air hotter than itself, is partly attributable to the copious perspiration and evaporation which then take place, and which absorb into the latent form the excess of heat then existing. Perspiration, both from the skin and internal surface of the lungs, occurs generally in proportion to the excess of heat. Dogs and other animals, when much heated, as they cannot throw off or diminish their natural covering, increase the evaporating surface by protruding a long humid tongue.

"The power in animals of preserving their peculiar temperature, has its limits. Intense cold coming suddenly upon a man who has not sufficient protection, first causes a sensation of pain, and then brings on an almost irresistible sleepiness, which, if indulged, would be fatal. Sir Joseph Banks having gone on shore one day near the cold Cape Horn, and being fatigued, was so overcome by the feeling mentioned, that he entreated his companions to let him sleep for a little while. His prayer granted, might have allowed that sleep to come upon him which ends not, the sleep of death! as, under similar circumstances, it came upon so many thousands of the army which Bonaparte led into Russia, and lost there during the disastrous retreat through the snows. Bonaparte's celebrated bulletin allowed that in one night, when the thermometer of Reaumur stood at nineteen degrees below zero, 30,000 horses died! Cold in inferior degrees, and longer continued, acting on persons imperfectly protected by clothing, &c., induces a variety of diseases, which destroy more slowly. A great excess of heat, again, may at once excite a fatal apoplexy; and heat in inferior degrees, but long continued, may cause those fevers, &c. which prevail in warm climates, and which are so destructive to strangers in these climates.

"Each species of animal has a peculiar temperature natural to it, and in the diversity are found creatures fitted to live in all parts of the earth; what is wanting in internal bodily constitution being found in the admirable protecting covering which nature has provided for them; covering which grows from their bodies, with form of fur or feather, in the exact degree required, and even so as in the same animal to vary with climate and season. Such covering, however, has been denied to man; but the denial is not one of unkindness: it is the indication of his superior nature and destinies. Godlike reason was bestowed on man, by which he subjects all nature to his use, and he was left to clothe himself.

"The human race is naturally inhabitant of a warm climate, and the paradise described as Adam's first abode may be said still to exist over vast regions about the equator. There the sun's influence is strong and uniform, producing a rich and warm garden, in which human beings, however ignorant of the world which they had come to inhabit, would have their necessities supplied almost by wishing. The ripe fruit is there always hanging from the branches; of clothing there is required only what moral feelings may dictate, or what may be supposed to add grace to the form; and as shelter from the weather, a few broad leaves spread on connected reeds, will complete an Indian hut. The human family, in multiplying and spreading in all directions from such a centre, would find, to the east and west, only the lengthened paradise, with slightly varying features of beauty; but to the north and south, the changes of season, which make the bee of high latitudes lay up its winter store of honey, and send migrating birds from country to country in search of warmth and food, would also rouse man's energies to protect himself. His faculties of foresight and contrivance would come into play, awakening industry; and as their fruits, he would soon possess the knowledge and the arts which secure a happy existence in all climates, from the equator almost to the pole. It is chiefly because man has learned to produce at will, and to control, the wonder-working principle of heat, that in the rude winter, which seems the death of nature, he, and other tropical animals and plants which he protects, do not in reality perish, even as a canary-bird escaped from its cage, or an infant exposed among the snow hills. By producing heat from his fire, he obtains a novel and most pleasurable sort of existence; and in the night, while the dark and freezing winds are howling over his roof, he basks in the presence of his mimic sun, surrounded by his friends and all the delights of society; while in his storerooms, or in those of merchants at his command, he has the treasured delicacies of every season and clime. He soon becomes aware, too, that the dreary winter, instead of being a curse, is in many respects a blessing, by arousing from the apathy to which the eternal serenity of a tropical sky so much disposes. In climates where labour and ingenuity must precede enjoyment, every faculty of mind and body is invigorated; and hence the sterner climates form the perfect man. It is in them that the arts and sciences have reached their present advancement, and that the brightest examples have appeared of intellectual and moral excellence." (P. 157.)

The second section, "on Light and Optics," will be found very interesting and instructive.

The former volume of Dr. Arnott's "Elements" passed through four editions in this country, and was translated into several languages; and we have no doubt that its successor will meet with equal approbation and honours.

---

*A Practical Treatise on Diseases of the Genitals of the Male; with a Preliminary Essay on the History, Nature, and General Treatment of Lues Venerea.* By John Maddox Titley, M.D.—8vo. pp. 403. Herbert, London, 1829.

Dr. Titley commences his work with a preliminary essay on the history, nature, and general treatment of Lues Venerea. As it cannot be a matter of much importance to modern practitioners to ascertain precisely the period when this disease first made its appearance, we shall not enter further into the subject of the history of its origin, than to observe that Dr. T. makes out a very plausible case from his literary researches to support his belief that lues venerea was known to the Old World from very early times. Mr. Bacot* comes to a contrary conclusion: he thinks that, if there is any single historical fact that can be said to be proved, it is that of the origin of syphilis being referrible to the latter years of the fifteenth century. As it appears to us that this discussion is more calculated to display the literary acquirements of the respective disputants than to add to our useful information, we shall not even endeavour to reconcile these discordant opinions.

Dr. Titley next proceeds to inquire whether lues venerea, or any form of it, can be so discriminated as to constitute a nosological species; and he wishes it to be understood that, in discussing this question, whether lues venerea, or any type of it, can be considered as a specific disease, he proceeds upon the idea of a species, which prevails universally, and he thinks necessarily, in all the nosological arrangements of eruptive fevers, namely, that each has a distinct cause. "Though in smallpox, measles, plague, &c. we have types of each, varying considerably from each other, we consider all those types to be varieties, and not species, because we know and believe them to be owing to one primary cause, but modified by the constitution of the patient, or by some secondary, supervening, or contingent cause."

In the practical part of this work, it will be seen that several kinds of primary venereal sores are distinguished, and respective modes of treatment prescribed.

"This is, however, nothing more than an useful expedient to direct attention towards some striking symptomatic feature with which certain practice has been supposed best to correspond. These specified patterns of sores are in truth only the best substitutes we have for pathological criteria, and extend no further than to the first link of a disease. I have adopted them on limited, and perhaps I might say empirical ground, not as applicable to discriminate the whole train of a venereal attack, or as indicating the operation of any ascertained pathological cause. Such sores are, in my judgment, of the same species as sores found in other parts of the body, and originating in causes not venereal. They may be successfully treated on the same principles as we should apply in the treatment of ulcers in general, allowing something clearly to be due to the peculiar structure and functions of the parts concerned; so that we cannot sever these primary sores, or any of

* Treatise on Syphilis, p. 17.

them, from association with ulcers of other parts and other causes. Even the advocates of mercury now admit that they may be healed on general principles of treatment; they only assert that a taint is left, which will break out afterwards in secondary symptoms. But we have a step farther to go, which completely overturns the doctrine of a syphilitic ulcer or chancre. No form of primary sore yet described is any thing more than a pattern arbitrarily selected from an endless number and gradation of forms, so blended and confounded together, that no two appearances can be delineated, between which experience does not present sores with more or fewer of the characteristics of either; so that it is impossible to fix a limit where one species ends and another begins." (P. 26.)

Dr. Titley is opposed to the doctrine of Mr. Carmichael, that each primary ulcer, of four several characters, which he has described, is, without exception, or with very rare and doubtful exception, followed by symptoms (when such ensue) of certain determinate types. The opinions of the most experienced army surgeons, as Guthrie, Hennen, Rose, &c. are adduced to show that extensive practical observation leads to conclusions which are destructive to the peculiar views entertained by Mr. Carmichael. Mr. Bacot* very fully and satisfactorily argues this question: he is also opposed to the theory of Mr. Carmichael, of a variety of syphilitic poisons. After giving a brief sketch of the treatment adopted by the Portugueze surgeons, and of the experience of the English military surgeons of the non-mercurial plan of treatment,† Dr. Titley supports their conclusions by some powerful corroborations from practitioners of other countries.

An interesting document has lately been published in Sweden upon the subject of the comparative frequency of secondary symptoms of syphilis, after various modes of treatment, which is much in favor of the non-mercurial practice. During five years, 16,985 venereal patients were treated in the hospitals of that country. Of this number, 39½ per cent. were trusted solely to strict dietetic rules, and six weeks were generally found sufficient for the cures, if the symptoms were not very severe: secondary symptoms happened in the proportion of 7½ per cent. Local and other modes of treatment were ordered for 5½ per cent., and of these 7 per cent. had after symptoms. The mercurial treatment was adopted in 49⅛ per cent.: of cases of secondary symptoms there were 14 per cent. The fumiga-

* Treatise on Syphilis, p. 71.

† Vide London Med. and Phys. Journal, August 1829, p. 157; Review of Mr. Bacot's Treatise, in which this subject is fully considered.

tory treatment by cinnabar was employed in $6\frac{1}{4}$ per cent.: the relapses were 22 to 100.

"A line of practice very similar to that employed in the British military hospitals, is adopted by M. Fricke in the venereal cases admitted into the hospital at Hamburgh. Every patient is bled to the amount of from six to twelve ounces, and the operation is repeated if necessary. About half a drachm of Sulphate of Magnesia is then given every three hours, and continued until repeated evacuations are produced. If the bowels afterwards become constipated, or the ulcers heal slowly, the use of the same remedy is renewed. As an external application to the chancres, Goulard water, or two grains of Sulphate of Zinc in six ounces of distilled water, is employed. When the size of the sore is much diminished, and it is no longer painful, lime water is used. If either of these lotions cause pain or inflammation, it is to be still further diluted.

"Buboes are first treated by compression, and, if resolution cannot be promoted, they are opened with a bistoury, and afterwards dressed with dry lint. Condylomatous tumors are removed by the knife, or cauterized, and the wound dressed with the same lotion as for the chancres. The patients are kept upon very low diet, consisting of vegetables, bread, and *soupe à l'eau*, twice a day. If at the end of a few days the symptoms are not alleviated, a few doses of mercury, in small quantities, are given, and are found sufficient to effect a cure.

"The results obtained by this mode of practice are highly satisfactory. Chancres and buboes are speedily cured, and the cicatrices are by no means so evident as when mercury has been employed. Chancres, from three to four lines in diameter, are generally cured, in female patients, in from one to three weeks; rather a longer time is required in male patients. M. Fricke, who has the advantage of retaining patients thus treated under his observation, has not yet observed any secondary symptoms." (P. 57.)

From these quotations, Dr. T. desires only to maintain the fact that every description of venereal disease may be cured without mercury, as certainly as by the aid of that medicine. The question whether either mode of treatment is to be preferred on any other ground than that of certainty, remains for subsequent consideration. Mr. Charles Bell* attempted, upon very weak grounds, to prove the danger of the non-mercurial practice. His opinions are referred to by Dr. Titley, for the purpose of showing that the cases adduced by Mr. Bell "do not bear, in the slightest degree, upon the question whether the venereal disease ought to be treated by mercury or not, or disprove the as-

* Surgical Observations, No. 5.

sertion that all simple venereal affections may be cured without it."

Having shown that mercury is in no sense a specific for any venereal disease, Dr. T. further maintains that lues is not a specific disease; inasmuch as its probable cause cannot be separated, by any sure distinction, from the cause of those affections, described under the denomination of pseudo-syphilis, and which have been believed to be produced independently of sexual intercourse.

Courses of mercury are objected to, but the author admits the utility of this medicine as an occasional alterative; or in some cases of fever, especially in a tropical climate, where he has seen large doses of calomel act like a charm; or in instances of acute inflammations, which demand an instant and powerful counter-agent. We perfectly agree in the estimate Mr. Bacot has formed of the advantages of the non-mercurial treatment, and we believe with him that, in a vast majority of cases, mercury is our sheet-anchor in the treatment of the venereal disease.* No abstract rules can be laid down for the precise doses or forms of this remedy: they must depend in every case upon the effects produced, and upon the constitution of the patient. That the various evils enumerated by Dr. Titley have arisen from the employment of mercury, may be very true; but we believe, from extensive experience in public and private practice, that, provided the remedy be *judiciously* employed, it is both harmless and useful. We certainly would not restrict the use of mercury by the narrow limits assigned by Dr. Titley, nor would we administer it in the incautious and hazardous mode which was once deemed necessary for the eradication of the venereal disease; but we are convinced that mild courses of mercury are very frequently imperatively necessary

Part I. "Diseases of the Penis." Amongst the various diseases to which the penis is liable, there are certain determinate kinds of ulceration, which have their origin in sexual intercourse, or in the direct application of some morbific matter secreted by the genitals. There are, again, other diseases of the male organ which may or may not originate in sexual commerce; as, for example, phymosis and paraphymosis: and, lastly, the penis is subject to various diseases, in common with other parts of the body, and having no reference to venereal congress. Dr. Titley

* Bacot on Syphilis, p. 60; and review of the work, London Med. and Phys. Journal, p. 159, August 1829.

proposes to follow this arrangement in his subsequent detail; but he first briefly alludes to those parts of the organization of the penis which materially modify the appearance and progress of disease at this part.

Of the diseases which originate from sexual intercourse, the first form described is the elevated ulcer, than which there is no species of sore so frequently seen on the genitals.

"It is usually found either on the glans penis, or upon the internal or external part of the prepuce, (in which situation it frequently induces phymosis;) but it may also occur on any of the neighbouring surfaces, as on the scrotum, thighs, &c.

"The first indications of the disease, usually in a few days after sexual intercourse, are itching and redness; then a small pustule is formed, with more or less inflammation; over which, after two or three days, a thin crust or scab appears. The confined matter under the scab occasions considerable pain, until it is released by the bursting of the cuticle, or by a portion of the scab being otherwise detached. After each effusion the scab becomes enlarged by accession of concreted matter: at first the scab is yellow, but afterwards becomes gradually darker, until it is nearly black, the colour being deepest at the centre. In this state, about four or six days after the first symptoms, the disease is usually submitted to the inspection of the surgeon.

"From the fourth to the sixth day, the figure of the elevated ulcer, as it next appears, after entire removal of the scab, is oval or circular, and concave. The colour of the concavity is glossy brown, unhealthy red, or more frequently dusky or yellow.

"When situated on the angle between the glans penis and the prepuce, this ulcer has frequently a deeper concavity, accompanied for a short time by hardness of the surrounding texture. When situated on the inner surface of the prepuce, the frænum, or any part behind the glans, except that just spoken of, it generally has but little concavity.

"About the eighth or ninth day, the ulcer is marked by elevation of the whole surface: at length it assumes the appearance of a fungus, raised so as to project, in a body, beyond the level of the surrounding skin. The outer edge of the elevated body often protrudes still further beyond the surface of that body, and the whole then appears to be of considerable thickness, and, when examined only by the eye, would give an idea of hardness. The outer edge is seldom lower than the elevated surface. The whole projection is often very considerable, especially when the ulcer is situated on the external surface of the prepuce, on the body of the penis, and on the scrotum. On the scrotum, so great is the elevation that it often resembles a large wart.

"When the ulcer is situated on the internal surface of the prepuce, or on the angle between the glans penis and prepuce, the projection is not so remarkable; but, even at this part, that pathognomic criterion is seldom dubious after the ninth day; when, by retracting the prepuce to its full extent, the elevated body cannot fail to be noticed at some point of the circumference.

"If the glans penis and internal prepuce be simultaneously ulcerated, a remarkable difference of appearance will be perceived in these parts at the period of fungus. On the prepuce the projection will be manifest; while on the glans penis a concavity will remain, and there the ulcer will rather continue an ulcerating progress than exhibit any granulations: when granulation has commenced, it will heal without any effort to project." (P. 77.)

The elevated ulcer has usually spread to its greatest extent from the eighth to the tenth day, and from the fourteenth to the eighteenth day has usually attained its greatest elevation. When it has attained its greatest height, it remains without variation for an uncertain period, after which it gradually, though very slowly, declines and heals. "To distinguish the elevated ulcer from other sores incident to the genitals, we have a sure criterion after the eighth day, in its elevated fungus, with an edge either higher or not lower than the surface of the fungus." Dr. Titley is of opinion that, in treating the elevated ulcer, a cure cannot be much accelerated, because the disease has a pretty well defined course.

"In its earlier stages, indeed, any interference, except for the purpose of allaying irritation, is commonly productive of mischief, and it is only after the ulcerative process has ceased, and the fungous state is established, that any thing can be done to accelerate a termination. During the state of pustule, means must be taken to protect it from friction; the bowels must be kept open; and, should there be much inflammation, (a circumstance which does not frequently occur,) the ulcer and neighbouring surface may be kept cool with a dilute lotion of subacetate of lead, or the spirit lotion. When the scab is forming, if there be much pain, a warm poultice will generally afford relief, and, when there is much irritation, it may be continued till the fungous state begins: it is sufficient to remove the scab by poultice or soft dressings, and the pain soon ceases; after which, spermaceti ointment will prevent the formation of scab, and, consequently, the recurrence of pain. If the scab be small, and there be no pain, it is best not to interfere." (P. 80.)

From friction of the clothes in walking, &c., inflammation, swelling, and phymosis, often supervene. As precautions against these, rest, abstemiousness, and attention to the state of the bowels, are necessary. During the states of pustule and scab, it is essentially beneficial to confine the patient to bed. Thus bubo will be often prevented, or, should it be formed, resolution be much more probable; local irritation will be avoided or lessened, and constitutional affections frequently prevented. If there be much symptomatic fever, the antiphlogistic treatment must be pursued. During the fungous or declining states, the surface of the sore may be touched daily with sulphate of copper, "but so lightly as to stimulate merely, and not prove an escharotic." "The employment of mercury will often retard the healing of this ulcer, and keep it obstinately stationary for several weeks; and in many instances this medicine is positively injurious, causing the ulceration to extend rapidly."

The superficial ulcer is not of very frequent occurrence: Dr. T. believes it is not found except on the external and internal surface of the prepuce.

"It is preceded by a pustule, the contents of which, on escaping, form a scab, and, as the scab becomes extended, the cuticle disappears from beneath it. The figure of the superficial ulcer is circular, or approaching to circular. The extent varies: in ordinary cases the largest size is about that of a shilling, but considerably greater if neglected or illtreated, especially when the constitution is much disturbed. The colour is a lively red, and the surface is on a level with the skin of the surrounding part.

"When healing is in progress, the surface becomes gradually depressed, and the cavity is not subsequently filled up, but remains after the ulcer is healed. Occasionally, for a short time, a fungus rises as from the elevated ulcer, projecting beyond the level of the surrounding skin." (P. 83.)

The distinctions between the superficial ulcer and the elevated ulcer, the indurated ulcer of the internal prepuce, herpes præputialis, and simple excoriations, are pointed out.

"In the treatment of the superficial ulcer, it is most necessary to observe the constitutional symptoms which are intimately connected with the progress of the sore, and, these being of a highly inflammatory type, require to be controlled by decisive measures. By such proceeding, in this, as in the case of elevated ulcer, we not only accomplish an earlier local cure, but, in all probability, often avert consecutive disease, or at least moderate the severity of it. * * *

"The constitutional disorder is most effectually subdued by bleeding, purgatives, and diaphoretics, in measure corresponding with the symptomatic indication. The local applications should be of a sedative kind during the inflammatory stage: a dilute lotion of subacetate of lead applied to the surface will generally be found most efficacious. If the sore become indolent, sulphate of copper, nitrate of silver, nitric acid, or the ointments of nitrate of mercury or subacetate of copper, and other stimulants, may be applied with advantage." (P. 85.)

The indurated ulcer is not of very frequent occurrence, at least within the range of Dr. Titley's observation and inquiries. It is found only on the internal prepuce and glans penis. "In treating the indurated ulcer, when unattended by violent symptoms of general disturbance, extirpation of the hardened portion by the knife is at once the shortest and the surest method." If the constitutional disturbance run high, and no counter-indication forbids, general bleeding should be at once adopted, as the only resource on which sole dependence can be placed. Purgatives, diaphoretics, and the local application of refrigerating lotions, should be conjoined, together with confinement to bed, and a free circulation of cool air. "Mercury in this description of sore is generally either hurtful or useless: constitutional symptoms do not commonly follow when this medicine has not been given."

The callous ulcer seems to have been more prevalent than any other species during the period of Mr. Hunter's practice; but, as Mr. Abernethy remarks, it is now so much less frequently met with, that many practitioners doubt whether there ever was such a disease as described by Mr. Hunter. Dr. T., however, has witnessed many examples of it, coinciding with his very good description, and completely vindicating his well-earned credit for accurate observation. For a description of this ulcer we must refer to the work. In the treatment of the callous ulcer, caustics have been employed, and, in Dr. T.'s opinion, they are highly useful, under certain limitations; but great discretion is necessary in the use of them.

"When the affection is recent, and we have fair grounds to hope that contamination has not been spread, the disease may be extinguished at once by caustic used with discreet freedom. On the contrary, when ulceration is completely established, caustics only produce aggravated evil, since the stimulant power of them urges mischief along the course of the lymphatics; and bubo, in such case, is very frequently a consequence.

"Nitrate of silver, which is very commonly used for this purpose, seldom destroys the entire sore; the inflammation which it produces increases the activity of the absorbents, and bubo is very often a consequence. A solution of sulphate of copper, on the contrary, effectually destroys the sore without producing irritation: this remedy should be employed in the proportion of a scruple of the salt to an ounce of distilled water, and is best applied on a pledget of lint. Absolute rest should be enjoined. If any redness be excited by the sulphate, a dilute lotion of subacetate of lead should be used, and a purgative dose of calomel given in the evening, with a requisite quantity of sulphate of magnesia in the morning. If healing does not readily take place, then the ulcer should be touched with a camel's-hair pencil dipped in a solution of sulphate of copper, in the proportion of ten grains of the sulphate to an ounce of water." (P. 93.)

Mercury may, and perhaps should, says Dr. T., be prescribed in the treatment of this ulcer. It promotes absorption of the hardened edge, excites the languid action of the part, and accelerates the healing of the sore. If the stomach is irritable, mercurial frictions are to be ordered; but, when the state of the stomach does not contra-indicate the internal use of the medicine, the bluepill in moderate doses, conjoined with small doses of opium, is recommended. The continuance of this treatment is to be defined by the sensible effects produced. A gentle mercurial action is to be sustained. "The horrible practice of profuse salivation" is properly condemned. "There is considerable difficulty in treating the callous ulcer when it occurs at the orifice of the urethra, since it is acted upon by the urine, and so prevented from healing. A short gum élastic catheter should be introduced and worn, and the part defended by a mucilaginous lotion, or mild ointment spread on lint."

Dr. Titley is convinced that much evil results from too meagre a diet when a medicine is employed which makes such large demands on the powers of the body as mercury does. He would allow two or three glasses of wine daily, unless there be some obvious counter-indication. If the patient have been a free liver, his ordinary stimuli must be moderately continued. From irregularity of life, the callous ulcer will often, instead of its usual indolence, exhibit great irritability. Here mercury must not, on any account, be used: "the whole virile member has often been destroyed by mercury exhibited under these circumstances." A soothing plan of treatment must be adopted.

"The patient should keep the recumbent posture, with the penis and testes well supported; and the use of poppy fomentations and poultices will be found the best local means of subduing irritation. A lotion composed thus, Powder of Opium ʒi. Lime water ℥viij. mix and strain, and to the strained liquor add mucilage of Gum Arabic ℥i., is also very effectual, and may be applied either warm or cold, as may be found best to agree with the feelings of the patient.

"After purging, opium should be given in repeated doses, combined with a saline, such as the liquor of the Acetate of Ammonia.

"In some cases it is necessary to make a decisive impression on the sanguiferous system, by abstracting blood from the arm; to reduce local inflammation by applying leeches, and to adopt a completely antiphlogistic method. Thus the affection may often be arrested at once, and most destructive gangrene prevented. In general, however, we have to contend with constitutions that will not bear the loss of blood: in these cases, therefore, we must resort to the tranquillizing influence of opium, in conjunction with saline diaphoretics, and at the same time sooth the irritated part by emollient applications.

If the sore have already put on a sloughing appearance, and there be not much surrounding inflammation, the lotion of dilute nitric acid* may be applied as a gentle stimulus, or poultices composed of the grounds of stale beer. Where the slough is large, and the parts at the same time sluggish, not evincing any sufficient effort to detach it, warm spirits of turpentine may be applied." (P. 97.)

If gangrene evidently results from deficiency of vital power, tonic means will be requisite, such as quinine with opium, diffusible stimuli, ammonia with musk or opium. Spirits, wine, or porter should be advised, with reference to the habits of the patient. In several cases we have had evidence of the truth of the following remark.

"It happens, not unfrequently, that a large sloughy sore, attended with a high state of inflammation and acute pain, and with corresponding constitutional disturbance, is rendered mild and tractable by a copious bleeding. This effect is often produced by a spontaneous effusion ensuing on the ulceration of vessels to which the sore has reached; when, in a few days afterwards, the slough separates, and the surface assumes a more healthy aspect." (P. 98.)

The subjects next discussed are the phagedæno-gangrenous ulcer, the sloughing ulcer, and buboes.

In the third chapter are described the various forms of secondary venereal affections. The treatment recommended is judicious.

Phymosis, Paraphymosis, and Excoriations, form the subject of the succeeding chapter, under the head of "Diseases which may or may not originate from sexual intercourse."

In the fifth chapter, Dr. T. treats of the different diseases which do not originate from sexual intercourse.

The second part of the work contains a good practical description of Gonorrhœa and Strictures of the Urethra. Diseases of the Scrotum and Testicles are next described.

The surgical student will obtain much valuable information from Dr. Titley's treatise, and to the practitioner the work will be found very useful, in cases of difficulty, as a book of reference.

* This is prepared by adding one drachm of pure nitric acid to a pint of water. The strength may be varied according to circumstances.

